# The Impact of School and After-School Friendship Networks on Adolescent Vaccination Behavior

**DOI:** 10.3390/vaccines8010055

**Published:** 2020-01-29

**Authors:** Daniele Mascia, Valentina Iacopino, Emanuela Maria Frisicale, Antonia Iacovelli, Stefania Boccia, Andrea Poscia

**Affiliations:** 1 Department of Business and Management, LUISS Guido Carli University, 00197 Rome, Italy; dmascia@luiss.it (D.M.); viacopino@luiss.it (V.I.); 2 Local Health Authority (ASL) Roma 1, 00193 Rome, Italy; 3 Sezione di Igiene, Istituto di Sanità Pubblica, Università Cattolica del Sacro Cuore, 00168 Rome, Italy; stefania.boccia@unicatt.it; 4 Local Health Authority (ASL) Roma 2–Ex RMB, 00157 Rome, Italy; 5 Department of Woman and Child Health and Public Health–Public Health Area, Fondazione Policlinico Universitario A. Gemelli IRCCS, 00168 Rome, Italy; 6 Facoltà di Medicina, Università Cattolica del Sacro Cuore, 00168 Rome, Italy; andrea.poscia@sanita.marche.it; 7 UOC ISP Prevention and Surveillance of Infectious and Chronic Diseases, Department of Prevention, Local Health Authority (ASUR–AV2), 60035 Jesi, Italy

**Keywords:** vaccination, adolescents’ health, social network analysis, LRQAP

## Abstract

Psychological and social characteristics of individuals are important determinants of their health choices and behaviors. Social networks represent “pipes” through which information and opinions circulate and spread out in the social circle surrounding individuals, influencing their propensity toward important health care interventions. This paper aims to explore the relationship between students’ vaccination health choices and their social networks. We administered a questionnaire to students to collect data on individual students’ demographics, knowledge, and attitudes about vaccinations, as well as their social networks. Forty-nine pupils belonging to 4 classrooms in an Italian secondary school were enrolled in the study. We applied a logistic regression quadratic assignment procedure (LR-QAP) by regressing students’ positive responsive behavior similarity as a dependent variable. LRQAP findings indicate that students’ vaccination behavior similarity is significantly associated with after-school social ties and related social mechanisms, suggesting that pupils are more likely to share information and knowledge about health behaviors through social relationships maintained after school hours rather than through those established during the school day. Moreover, we found that vaccination behaviors are more similar for those students having the same ethnicity as well as for those belonging to the same class. Our findings may help policymakers in implementing effective vaccination strategies.

## 1. Introduction

Emerging debate on the general level of vaccinations’ acceptance is widespread across developed countries. A large movement against vaccination is becoming greater and greater through traditional media channels, social media platforms, and online communities [1,2]. As such distrust grows, countries are required to face a new social challenge, whose negative consequences may lead, progressively and dangerously, to an erosion of the general level of populations’ protection and to the reappearance of “old fashioned” pandemic diseases [3]. In response to this trend, many initiatives have been promoted by decision makers to reinforce the obligation to vaccinate [4,5].

The World Health Organization (WHO) Strategic Advisory Group of Experts (SAGE) on immunization uses the term “vaccine hesitancy” to indicate the individual’s propensity to refuse some or all vaccines as established by clinical guidelines and schedules [6,7]. Motivations underlying the scarce propensity to receive vaccination have relevant behavioral foundations. Prior studies documented that individual attributes, such as age, gender, ethnicity, health status, behaviors, and perceptions directly impact vaccination attitude in large communities [8,9], as well as specific categories of individuals, such as adolescents [7,10], adults and the elderly [11,12], health workers [13], and homosexuals [14]. Behavioral determinants of a scarce propensity to receive immunization are grounded in the general idea of invulnerability in individuals, who, at least for some infectious diseases, feel themselves healthy and strong enough to successfully solve their health problems without any immunization protection. Moreover, the high level of distrust toward the side effects of vaccines and the perceived economic interests of pharmaceutical companies in creating new medical needs are perceived as strong reasons for refusal. Other factors that likely affect vaccine hesitancy and influence behavior are (i) confidence, i.e., trust in the effectiveness and safety of vaccines as well as in the health care system and in the motivations of policy–makers involved; (ii) complacency that indicates the perception of individuals about the risks of vaccine-preventable diseases along with the relative need of vaccination; and (iii) convenience, which concerns all factors influencing the access to vaccines such as availability, affordability, accessibility, health literacy, and the appeal of immunization services [6].

Social and psychological circumstances may need to be considered to implement successful preventive strategies. Particularly, research on adolescents’ behavior and development is crucial to describe the different reactions to immunization programs. Such relevance is motivated by twofold reasons, both connected to the extraordinary changes they experience during this phase. First, during the early adolescence (11–14 years old), they undergo many vaccinations against infectious diseases, such as the HPV immunization and recalls to tetanus, diphtheria, and pertussis. Second, adolescence represents the time span when consciousness, responsibility, and awareness toward healthy behaviors and health prevention emerge. Recent studies highlighted the role of parents’ networks in addressing children vaccination choices, arguing that the influential power of networks is a greater predictor than any other individual characteristic [15]. In other words, parents play a key role in vaccine acceptance, but the immunization choices for their children are not taken individually. Rather, they are a product of an influential path where health care providers, family members, and friends act as sources of information, direction, and advice [15].

Although it is undeniable that parents and family concur to build a personal identity, particularly in a period when the young individual is not fully developed, children and their way of living have greatly changed over time. Such changes have affected, in turn, their degree of freedom in making decisions. This is even truer in the case of health choices, often displaced in an intimate and personal sphere, such as sexuality [16].

Schools are primarily involved as social structures where young individuals establish and develop network ties and, given their educational function, they are eligible settings for health promotion interventions [17]. Given the mutual dependency between social interactions and health behaviors, friendships are identified as an important element for adolescents’ health [18]. Prior studies documented the importance of adolescents’ network in the promotion of healthy choices, such as the propensity to physical activity [19] or the tendency to obesity [20]. Scholars found an association between the similarity (homophily) in certain health statuses and friendship networks, confirming the occurrence of contagion phenomena among young individuals [21,22,23,24]. These homophilous behaviors can be explained by the process of friendships’ selection [23,24] or, conversely, by the sharing of habits among friends [25]. Other studies analyzing the role of friendship networks in adult obesity or smoking cessation [26,27] have pointed out the necessity to grow preventive strategies, addressing health behaviors, that consider the relational context of individuals [28]. However, few studies analyzed the role of social networks in health promotion topics, such as immunization behavior, and no exhaustive conclusions are available about vaccination hesitancy. According to the available knowledge, social networks allow a greater social exposure to medical information, which, in turn, raises people’s perceptions of the benefits of immunization [29]. Moreover, imitation (contagion) effects among peers and community members may lead to similar decisions about immunization [30].

Although the role of social communities in shaping vaccination preferences and attitudes is already recognized, less is known about where such preferences and attitudes frequently emerge.

The aim of this study is to understand factors affecting the vaccination behavior of adolescents. Specifically, we aim to explore the relationship between pupils’ positive responsiveness to vaccination and their friendship networks maintained during school hours (school network) and outside school hours (after-school network).

## 2. Materials and Methods

### 2.1. Research Setting

This study is part of the larger Italian research project “VacciniAmo le Scuole” aimed at encouraging the promotion of vaccination and primary prevention across the Italian educational system [31].

In Italy, routine immunization programs, including poliovirus and diphtheria–tetanus have been mandatory since the early 1960s. The Hepatitis B vaccine was added only in 1991, with the introduction of a universal vaccination for newborn and children up to 12 years. As the reduction and eradication of vaccine-preventable diseases have become a paramount priority in the public health agenda, other vaccinations, even if not mandatory, are free of charge for all the individuals in the country, strongly recommended by the Italian Ministry of Health and included in the health benefit packages (LEAs). According to the process of decentralization started in the late 1990s in Italy, regions are required to set up their vaccination strategies, which must be consistent with the National Vaccination Plan [32]. Public facilities are in charge of providing vaccination to children, which are usually administered within specific departments of the Local Health Authorities (LHAs). According to this organizational setting, pediatricians and General Practitioners (GPs) run counselling activity to their communities and families about vaccines [33]. Moreover, a prominent role in health promotion activity is played by schools. This justifies our study framing and the research setting chosen to run data collection [17].

### 2.2. Data Collection and Participants

According to the project’s objectives, a 90-min training program about vaccination was carried out in all four classrooms of a secondary school located in the central Italy. During the training, background information about vaccines was provided by public health physicians, and a role-playing activity allowed participants to experience practical situations about immunization. The training gave the opportunity to administer a structured paper questionnaire to the attending students. Before the training, pupils’ parents received (at home) a similar structured paper questionnaire together with the request for the informed consent to allow the data collection.

These questionnaires represent the primary data source for this study. Questionnaires submitted to students and their parents (one respondent per each pupil) slightly differed and were structured in three different sections. The first section collected demographic characteristics of the respondents and, only for pupils, specific questions about the school and after-school friendship networks. Particularly, students were asked to list up to 8 school mates—both within and outside their own class—with which they interact the most both during school and after-school hours. The second part of the questionnaire attempted to appreciate the level of knowledge and awareness about immunization. The third part of the questionnaire was aimed at collecting the attitude and feelings toward vaccinations in both the categories of respondents.

In order to measure the pupils’ responsiveness toward vaccination, data on students’ vaccination profile were collected by the Local Health Authorities to which the school administratively belonged after one month since the delivery of the training program at the school.

### 2.3. Data Analysis

As our goal is understanding how friendship ties may affect the responsiveness toward vaccination, our data are relational [34]. Social network analysis (SNA) techniques were applied to understand the role of friendship networks in determining pupils’ vaccination positive response behavior. SNA comprises a set of tools and methods to investigate the relational data [34], supporting researchers in understanding the role of social relations in individual or organizational choices. In particular, we applied multiple regression quadratic assignment procedures (MRQAPs), a statistical technique widely used in previous social network research to explore factors associated with the formation of network ties [35,36] as well as the adoption of similar behavior [37].

MRQAP tests aim to offer an alternative to traditional statistics for inference. MRQAPs are permutation tests for multiple linear regression model coefficients when data are organized in square matrices of relatedness among n objects and when the unit of analysis is the dyad. Such a data structure is typical in social network studies, where variables indicate some type of relationship between a given set of actors [38]. The MRQAP test, well-known as “double semi-partialing” or DSP, has been proved to show robustness under all multicollinearity conditions and thus solve the problem of the structural autocorrelation of network data [39].

One important implication of this methodology is that all covariates have to be transformed into pseudo-network data. In our case, all the covariates represented individual attributes and preferences of pupils as measures of differences/similarities among each pair (dyad). Practically, continuous covariates (i.e., parent’s age) entered the models as absolute differences between pupil *i* (sender) and pupil *j* (receiver). In this case, the smaller the difference, the greater the similarity (homophily) in the dyad. On the contrary, categorical covariates (i.e., pupils’ attending class) or binary covariates (i.e., gender) were transformed as binary variables describing the “match” between the two, assuming a value of “1” if both pupils in the dyad were in the same category or a value of “0” otherwise.

In this paper, we used the logistic regression quadratic assignment procedure (LRQAP), a specification of the multiple regression quadratic assignment procedures family [35,36], which is best suited for binary networks and variables. Given the binary nature of our dependent variable, namely, the similarity in positive responsiveness, LRQAP was adopted to produce estimates of our regression models (in order to test the robustness of our findings, we conducted additional MRQAP tests and the results of this supplementary analysis, consistent with those present in this paper, are available from the authors upon request).

All analyses were performed using the UCINET 6.598 software package [35].

The school and after-school friendship network data collected were described in one-mode sociomatrices aimed at appreciating the inter-personal ties among pupils.

### 2.4. Dependent Variables

“Positive Responsiveness”. The dependent variable in our study describes the similarity in the responsiveness to the training program pupils have been involved in and that occur in each dyad. In particular, the variable describes the presence of a positive reaction in terms of new vaccinations observed in the month that followed the training intervention. This information was collected through the pupils’ vaccination profile observed at the relevant Local Health Authority and was measured as a binary variable assuming the value of “1” when the pupil positively reacted to the training intervention and accepted being vaccinated in the month after and a value of “0” otherwise. Then, we built a squared matrix in which pupils are reported in rows and columns, and interception cells indicate the occurrence of similarity in their positive reaction to the training intervention (“1” if it was recorded, “0” otherwise).

### 2.5. Explanatory Variables

#### Network Measures and Social Mechanisms

“School Friendship Network”. In the questionnaire, pupils were asked to name the classmates with whom they talk the most and usually spend time with during school hours. Using this information, an adjacency matrix representing these interpersonal relations among children was built up. The matrix indicated both in rows and columns the pupils enrolled in the study and in the cells, the value of “1” when a friendship relation occurred, with a value of “0” otherwise.

“After-School Friendship Network”. Students enrolled in the study were asked to list the classmates with whom they talk the most and they usually spend time with after school hours. As in the case of the school friendship networks and according to the information collected, we built an adjacency binary matrix representing the friendship among children outside school. In the matrix, the pupils enrolled in the study were observed both in rows and columns, and the presence of their relation was reported using binary values in the cells.

“Social Mechanisms.” In order to investigate the social processes underlying the configuration of the two friendship networks observed, three different social mechanisms have been taken into consideration in our models. First, given the positive emotions generally involved in a friendship, we considered the *reciprocity* of pupils’ relational ties. Particularly, reciprocity indicates the likelihood of individuals to reciprocate relations and thus to be mutual in the social exchange process. Second, we considered the networks’ *closure*, which refers to the natural tendency of individuals to triangulate in their networks and, eventually, to connect and cooperate within small groups. Third, a measure of *preferential attachment* was used in our models. Specifically, the preferential attachment indicates the likelihood of actors to link to others depending on their level of popularity. In practical terms, the preferential attachment indicates the extent to which an actor is more likely to become progressively more popular as an effect of his/her centrality [40]. We specified and included these three social mechanisms for the two friendship networks tested in our models.

### 2.6. Control Variables

“Pupils’ Ethnicity”. Prior studies documented that some forms of homophilous behavior may be predicted by the homogeneity in the racial ethnicity [41]. Thus, we considered a binary variable indicating the pupils’ racial ethnicity. This information allowed us to derive a similarity matrix assuming in both rows and columns the pupils and in the cells, the value of “1” whether pupils in the dyad were found to be similar in terms of this attribute and a value of “0” otherwise.

“Pupils’ Gender”. Since we were interested in investigating any form of gender homophily in our sample, we considered in our models pupils’ similarity in the gender attribute. Considering the related binary variable, we built up a matrix assuming the value of “1” whether the pair of actors in the dyad belonged to the same gender and a value of “0” otherwise.

“Pupils’ Attending Class”. We considered in our models the class attended by pupils within the school. A categorical variable aimed at appreciating this attribution information allowed us to derive a squared matrix representing in both columns and rows the pupils and in the cells the value of “1” whether they belonged to the same class, and a value of “0” otherwise.

“Pupils’ Perceived Relevance of Vaccination”. In the questionnaire, children were asked to declare their perception about the relevance of vaccines in the prevention of infectious diseases. This perception was operationalized by a 7-point Likert scale ranging from “1” (not relevant at all) to “7” (very relevant). Because of the categorical nature of this variable, we built up a matrix describing the absolute difference in the perceived relevance between the pair of actors in each dyad.

“Parents’ Perceived Relevance of Vaccination”. Prior studies documented that parents’ individual characteristics may predict the responsiveness to vaccination [7]. As we collected information from parents about their perceived relevance of immunization in the prevention of infectious diseases, we included such information in our models by a matrix describing the absolute difference in the perceived relevance between the pair of parents in each dyad.

“Parent’s Age”. Similarly, we chose to consider parents’ similarity in terms of age as a potential predictor of the similarity in the responsiveness to vaccination. Since this variable is continuous, we calculated the absolute difference in age for responding parents’ pupils in the dyad.

“Pupils’ Medical Visits”. Similar behaviors in the access to the NHS system may predict positive responsiveness to the vaccination training program. Under this hypothesis, we considered in our models the average number of medical visits that pupils underwent every year, as self-reported by their respondent parent. In order to appreciate this variable, we described the degree of similarity in each dyad using a matrix, reporting both in rows and columns the name of pupils and in the cells the absolute difference in the number of visits occurring in the dyad.

## 3. Results

Out of 83 students attending the different classes, 49 pupils and their parents agreed to participate in the present study. All data pertaining to these students were collected and considered in our analysis (response rate was 60%). Table 1 shows the demographics of sampled pupils.

**Figure 1:**
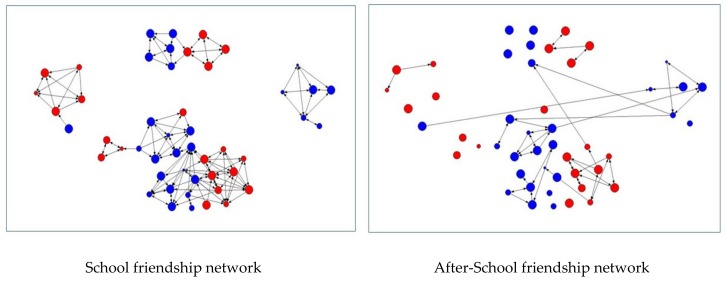
School and After-School Friendship Networks. Note: each node represents a pupil. The size of the node indicates the perceived relevance of immunization in the prevention of infectious diseases as declared by the children. The color of the nodes indicates pupils’ gender (red = females, blue = males).

We represented graphically the 49 pupils enrolled in the models by using the Netdraw software package [35]. Figure 1 describe the sociograms of the pupils’ school friendship network and after-school friendship network, both based on different attributes included in the models tested. In particular, the size of each node (pupil) indicates the perceived relevance of immunization in the prevention of infectious diseases as declared by pupils and its color indicates the pupil’s gender. Red nodes represent females, while blue nodes indicate males.

Table 2 shows descriptive statistics of the variables enrolled in our study as well as the quadratic assignment procedure (QAP) correlation results. “School friendship network” is positively and significantly correlated with social mechanisms underlying social ties, such as the “Reciprocity” (r = 0.607, *p* < 0.000(0)1) and the Closure (r = 0.633, *p* < 0.000(0)1). Similarly, “Closure” and “Reciprocity” during school hours are moderately correlated with each other (r = 0.527, *p* < 0.000(0)1). “After-school friendship network” is, in turn, associated with its own social mechanisms, such as “Reciprocity” (r = 0.586, *p* = 0.000(0)1). “School friendship network” is associated positively and significantly with reciprocal ties occurring after school hours (r = 0.462, *p* < 0.000(0)1). In addition, “after-school friendship network” is associated with the “school friendship network” (r = 0.473, *p* < 0.000(0)1) and the reciprocal ties during school hours (r = 0.462, *p <* 0.000(0)1). Similarly, the two measures of “Reciprocity” (both during and after school hours) are associated positively and significantly (r = 0.473, *p* < 0.000(0)1). The social mechanisms of “Preferential Attachment” both during and after school are moderately associated (r = 0.563, *p* < 0.000(0)1). The similarity in the attendance of pupils’ class is associated with “School friendship network” (r = 0.472, *p* < 0.000(0)1), “Reciprocity” (r = 0.472, *p* < 0.000(0)1), and “Closure” (r = 0.67 *p* < 0.000(0)1).

Three different models were tested in order to appreciate the statistical association among variables. Model 1 includes variables that represent social relationships that students maintain during school day as well as control variables. Model 2 includes after-school relationships and related social mechanisms as well as the other possible factors explaining vaccination behavior similarities Finally, Model 3 aims to test only the effect of control variables. 

Table 3 shows LRQAP findings. No statistical significance was found among the dependent variable and the school friendship networks and social mechanisms variables. Nevertheless, after-school friendship networks seem to be positively and significantly associated with the similarity in vaccination responsiveness (OR = 1.473), together with the reciprocity of pupils’ ties (OR = 1.837) and preferential attachment (OR = 1.341). These findings suggest that pupils are more likely to share information and knowledge about health behaviors through social relationships maintained after school hours rather than through those established during the school day.

According to the explanatory variables enrolled in our models, our results show that the similarity in racial ethnicity (OR = 6.130 in model 1; OR = 5.391 in model 2; OR = 5.983 in model 3) is positively and significantly associated with the similarity in vaccination behavior. Similarly, belonging to the same class seems to positively act as an antecedent of the similarity in vaccination responsiveness (OR = 1.836 in model 1; OR = 1.681 in model 2; OR = 1.820 in model 3). Finally, a negative and significant association was found between the similarity in pupils’ gender and the dependent variable (OR = 0.865 in model 1; OR = 0.836 in model 2; OR = 0.860 in model 3). Thus, vaccination behaviors are more similar for those students having the same ethnicity or diverse gender as well as for those belonging to the same class. Finally, no statistically significant association was found between the dependent variable and the similarity in the perceived relevance of being vaccinated expressed both by the pupil and his or her parent, in the frequency of his or her access to the general practitioners, and in parents’ age.

## 4. Discussion

In a historical period of economic, political, and social pressures, the debate on vaccination, as well as on the legitimate role of welfare states in the promotion and prevention of health [42], is extraordinarily hot. Behavioral mechanisms of trust regarding vaccination procedures underwent a gradual erosion. At the same time, a strong concern of the heterogeneity in the distribution of vaccination coverage is observed from country to country [43] and, in Italy, even from region to region. In a recent review of the vaccination regional plans (RPPs), Rosso et al. (2015) showed how such variability is not only in the outcomes, but also in the appraisal activity of regional plans, and the initiatives against unequal access to vaccine immunization are still far from an effective implementation [44].

To uncover this concern, it is necessary to consider the contextual factors affecting the policy and management decisions [45], such as laws and regulation and the level of integration between professionals and public authorities [46]. In this sense, previous studies have shown that financial incentives of doctors decisively encourage the immunization level in the reference population [46]. Finally, especially at this point, the importance of the media and the public in promoting a certain perception of vaccines may not be ignored [47]. Individuals and families are the preferential recipients of this communication flow, according to which they adjust their level of trust/mistrust toward vaccines and their consideration of the state as the principal health promoter [42].

The role of social networks in determining health choices has been robustly recognized in different context and communities. Assuming a micro-level perspective of analysis, our paper aims to disentangle the role of social networks, and, more specifically, of friendship ties, in the formation of vaccination behaviors among young adolescent attending secondary school in Italy. Our findings show that social ties between students are positively associated with their vaccination behaviors. More specifically, friendship ties occurring after school are more likely to create similarity in vaccination positive responsive behavior.

Although schools are recognized as ideal social contexts for health promotion activities, our results seem to be consistent with prior studies suggesting that more effective interventions are needed to foster the interaction between schools and the surrounding environment [17,47] and to translate planning objectives into genuine benefits for the target population [47]. In Italy, we may consider that schools have experienced profound changes. Several reforms have characterized the organizational life of these settings. Public policy interventions addressed in schools have been numerous and turbulent. Although health promotion has been always considered as a priority in the educational system, it is necessary to take into account the numerous challenges school managers have had to face in the recent past and present, such as job insecurity of teachers, lack of financial resources, and infrastructure inadequacy. Some critical points have to be addressed to effectively manage prevention programs, which should regard capacity building activities for project management, commitment, and leadership, as well as the availability of human, financial, material, and temporal resources [47]. Moreover, the WHO SAGE group recommends integration between health and non-health services through joint initiatives in order to address complacency and convenience hesitancy factors [6]. Our results also document the association between such similarity in positive responsiveness and certain individual attributes of pupils, such as their racial ethnicity and the class attended. The level of homophily in these characteristics seems to affect the attitude toward vaccination. Moreover, a diversity tendency (heterophily) has been observed in regard to gender. In other words, pupils are more likely to be similar in terms of vaccination responsiveness when they are different in terms of gender. This last result surely deserves more attention in future research. Understanding the network ties that classmates establish and maintain after school is of interest to implement effective vaccination strategies at organizational, regional, and national levels.

We believe that our findings are of interest from at least three different perspectives. First, our paper contributes to clarify the role of social networks in determining choices related to health issues and behaviors. Although several empirical studies demonstrated a strong association between the two, our study may represent a new original piece of evidence in the broader research topic of social networks and health care. Second, our results may have relevant methodological implication. The use of social network analysis may be of interest for those who are interested in exploring the mechanisms underlying the social structures of any community and setting. Third, our results may be helpful for managers at different level of decision making. The investigation of social structures could be useful to understand the spread of a disease within a population [48]. Moreover, policy makers and schools’ managers could project targeted vaccination campaigns using social network data [49].

Notwithstanding the important potential contributions that the paper may provide researchers and decision makers with, the study is not without limitations. Firstly, we observe that the present study is cross-sectional precluding the possibility to infer causal relationships among variables. Future longitudinal studies will be helpful to shed more light on how social selection and influential processes play a role in this context, explaining more widely how social networks affect the propensity of pupils toward vaccinations. Secondly, our study may have not considered other variables that can potentially help to unfold pupils’ attitude toward vaccination. Thirdly, the empirical context of this research is represented by one school in the Italian NHS. The limited research scale of our setting may significantly constrain the generalizability of our findings. Furthermore, we note that several factors that affected the intervention delivery when our survey was administered can help to explain the low response rate achieved. The most significant is probably the extensive spreading of the no-vax movement in the regional area where we conducted our research, which seemed to have influenced pupils enrolled for the study along with their parents, who significantly limited the consent given to their children for participation in the survey. Despite the above limitations, we believe that our analytical approach, once replicated at a larger scale in other health systems, could helpfully support policymakers’ interventions aimed at understanding vaccination problems.

## 5. Conclusions

The present study informs policymakers about the importance that school and after-school friendship networks have for the vaccination behaviors of adolescents. Adolescents’ attitudes and behaviors likely affect their health conditions and propensities toward the health system over time. Social networks created with classmates seem to be important in this vein because they help explain how individuals’ opinions regarding certain educational and health interventions take shape in scholastic contexts. Only the joint effort of policymakers in these two fields, education and healthcare, will improve our ability to tackle individual vaccination hesitancy as well as to implement more coordinated and focused health interventions. Moreover, understanding the role of social networks in shaping health behaviors may lead to effective health promotion campaigns that could be delivered in institutional or recreational places taking into account social ties. We can consider the present research as a first attempt to incorporate social network effects in the study of vaccine hesitancy and its determinants. If replicated at a broader scale, this study could better inform policymakers to promote strategies aimed at tackling vaccination hesitancy.

## Figures and Tables

**Table 1:** Descriptive statistics.

Characteristics of Surveyed Pupils (N = 49)	
Positive Responsiveness N (%)	
0 (No)	10 (20%)
1 (Yes)	39 (80%)
Pupils’ Ethnicity N (%)	
Italian	45 (92%)
Other racial ethnicity	4 (8%)
Pupils’ Gender N (%)	
Males	27 (55%)
Females	22 (45%)
Pupils’ Attending Class	
Class 1	18 (37%)
Class 2	12 (25%)
Class 3	9 (18%)
Class 4	10 (20%)
Pupils’ perceived relevance of vaccination	
1	0 (0%)
2	0 (0%)
3	2 (4%)
4	4 (8%)
5	10 (20%)
6	6 (13%)
7	27 (55%)
Parents’ perceived relevance of vaccination	
1	0 (0%)
2	0 (0%)
3	1 (2%)
4	3 (6%)
5	7 (14%)
6	2 (4%)
7	36 (74%)
Parent’s Age Average ± SD (Range)	43.53 ± 4.59
Pupils’ Medical visits	
1 = once per week (every 2 weeks)	0 (0%)
2 = once per month	5 (10%)
3 = once every 2–4 months	10 (20%)
4 = once every 6–9 months	28 (57%)
5 = once per year or never	6 (13%)

**Table 2:** QAP correlations.

	Variables Heading	1	2	3	4	5	6	7	8	9	10	11	12	13	14	15
1	*Positive Responsiveness*	-														
2	*School Friendship Network*	0.044 *	-													
3	*School Friendship Network* *—Reciprocity*	0.044 *	0.607 **	-												
4	*School Friendship Network* *—Closure*	0.068 *	0.633 **	0.527 **	-											
5	*School Friendship Network* *—Preferential Attachment*	0.001	0.161 **	0.048 **	0.191 **	-										
6	*After-School Friendship Network*	0.064 **	0.473 **	0.462 **	0.337 **	0.082 **	-									
7	*After-School Friendship Network* *—Reciprocity*	0.064 **	0.462 **	0.473 **	0.345 **	0.096 **	0.586 **	-								
8	*After-School Friendship Network* *—Closure*	0.058 *	0.374 **	0.354 **	0.349 **	0.103 **	0.317 **	0.317 **	-							
9	*After-School Friendship Network* *—Preferential Attachment*	0.161 **	0.09 **	0.05 **	0.113 **	0.563 **	0.146 **	0.105 **	0.177 **	-						
10	*Pupils Ethnicity*	0.308 **	0.035	0.035	0.026	0.113	0.029	0.029	0.067 *	0.164 *	-					
11	*Pupils Gender*	−0.007	0.193 **	0.193 **	0.135 **	−0.013	0.148 **	0.148 **	0.161 **	0.006	0.081 **	-				
12	*Pupils’ Attending Class*	0.110 **	0.472 **	0.472 **	0.64 **	0.067 **	0.244 **	0.244 **	0.228 **	0.034 *	0.005	−0.033 *	-			
13	*Pupils’ Perceived Relevance of Vaccination*	0.012	−0.019	−0.019	−0.007	−0.047	0.008	0.008	0.012	−0.022	0.086 *	0.022	−0.012	-		
14	*Parents’ Perceived Relevance of Vaccination*	0.099	0.033 *	0.033	0.024	0.006	0.012	0.012	0.037	0.028	0.108	0.018	0.044 *	0.048	-	
15	*Parent’s Age*	0.074	−0.056 **	−0.056 **	−0.052 *	−0.162 **	−0.07 **	−0.07 **	−0.056 **	−0.142 **	−0.061	0.033 **	0.000	0.022	0.149 *	-
16	*Pupils’ Medical Visits*	0.072	−0.042 *	−0.042 *	−0.005	−0.125 **	−0.028	−0.028	−0.032	−0.128 **	0.01	−0.021	0.039	−0.061	0.051	0.264 **

Significance levels: ** *p* ≤ 0.05, * *p* ≤ 0.1. All variables are at the dyadic level.

**Table 3:** Logistic Regression Quadratic Assignment Procedure (LRQAP).

Variables	Model 1	Model 2	Model 3
Intercept	−1.148 (0.317)	−1.574 (0.207)	−1.257 (0.285)
*Network Variables*			
*School Friendship Network*	−0.076 (0.926)		
*School Friendship Network—Reciprocity*	−0.108 (0.898)		
*School Friendship Network—Closure*	0.113 (1.119)		
*School Friendship Network—* *Preferential Attachment*	−0.033 (0.968)		
*After-School Friendship Network*		0.387 * (1.473)	
*After-School Friendship Network—Reciprocity*		0.608 ** (1.837)	
*After-School Friendship Network—* *Closure*		−0.050 (0.951)	
*After-School Friendship Network—* *Preferential Attachment*		0.293 ** (1.341)	
*Exploratory Variables*			
*Pupils’ Ethnicity*	1.813 ** (6.130)	1.685 ** (5.391)	1.789 ** (5.983)
*Pupils’ Attending Class*	0.607 ** (1.836)	0.519 ** (1.681)	0.599 ** (1.820)
*Pupils’ Gender*	−0.145 * (0.865)	−0.180 ** (0.836)	−0.151 ** (0.860)
*Pupils’ perceived relevance of vaccination*	−0.032 (0.969)	−0.018 (0.982)	−0.028 (0.973)
*Parents’ perceived relevance of vaccination*	0.109 (1.115)	0.100 (1.106)	0.108 (1.114)
*Parent’s Age*	0.046 (1.047)	0.059 (1.061)	0.048 (1.050)
*Pupils’ medical visits*	0.122 (1.130)	0.181 (1.198)	0.132 (1.141)
*# Observations*	2352	2352	2352
*# Permutations*	10,000	10,000	10,000
*Model p-value*	0.022	0.009	0.016
*R-squared value*	0.123	0.139	0.122

** *p* ≤ 0.05, * *p* ≤ 0.1; Odds ratio in parentheses.
